# Stem cell exosomes inhibit angiogenesis and tumor growth of oral squamous cell carcinoma

**DOI:** 10.1038/s41598-018-36855-6

**Published:** 2019-01-24

**Authors:** Leonie Rosenberger, Marcelo Ezquer, Fernando Lillo-Vera, Paulina L. Pedraza, María Ignacia Ortúzar, Paz L. González, Aliosha I. Figueroa-Valdés, Jimena Cuenca, Fernando Ezquer, Maroun Khoury, Francisca Alcayaga-Miranda

**Affiliations:** 1 Consorcio Regenero, Chilean Consortium for Regenerative Medicine, Santiago, Chile; 2 Centro de Medicina Regenerativa, Facultad de Medicina Clínica Alemana-Universidad del Desarrollo, Santiago, Chile; 3 Cells for Cells, Santiago, Chile; 4 Laboratory of Nano-Regenerative Medicine, Faculty of Medicine, Universidad de los Andes, Santiago, Chile

## Abstract

Recently, exosomes secreted by menstrual mesenchymal stem cells have been identified as inhibitory agents of tumor angiogenesis and modulators of the tumor cell secretome in prostate and breast cancer. However, their direct effect on endothelial cells and paracrine mediators have not yet been investigated. Using a carrier-based cell culture system to test the scalability for exosome production, we showed that different types of endothelial cells present specific kinetics for exosomes internalization. Exosome-treatment of endothelial cells increased cytotoxicity and reduced VEGF secretion and angiogenesis in a dose-dependent manner. Using the hamster buccal pouch carcinoma as a preclinical model for human oral squamous cell carcinoma, we demonstrated a significant antitumor effect of intra-tumoral injection of exosomes associated with a loss of tumor vasculature. These results address up-scaling of exosome production, a relevant issue for their clinical application, and also assess menstrual stem cell exosomes as potential anti-angiogenic agents for the treatment of neoplastic conditions.

## Introduction

Head and neck cancer, with oral squamous cell carcinoma as its major subtype, ranks among the ten most common cancer types worldwide^1^. Despite advances in treatment and diagnosis, its five-year survival rate is only around 50%^1,2^. The presence of metastases is the most important prognostic indicator of survival^3,4^ and depends on the formation and establishment of new blood vessels, a process known as tumor angiogenesis^3,5–7^. In fact, overall survival is reduced with hypoxic, angiogenic and vascular endothelial growth factor (VEGF)-expressing tumors^8,9^. Therefore, targeting tumor angiogenesis is a promising approach of cancer therapy in head and neck cancer.

Exosomes are small secreted membrane vesicles that mediate intercellular communication with a specific molecular content that is dependent on their parent cell’s signature^10–12^. Recent studies show that mesenchymal stem cell (MSC)-derived exosomes exert paracrine effects on angiogenesis^13,14^. Since the exosomal content is linked to the cell of origin^12^, it is not surprising that both pro- and anti-angiogenic effects of exosomes secreted by MSCs of different tissues are reported in the literature^15–19^. These opposing effects reflect the influence of the tissue-specific microenvironment on the exosomal cargo signature of MSCs and their biological function on target cells^20,21^. The endometrium harbors a specific type of MSC, termed menstrual mesenchymal stem cell (MenSC) that is shedded during menstruation^22–24^. This phase of the endometric cycle is characterized by an angiostatic environment associated with the expression of Thrombospondin-1, mainly during the secretory phase which includes menstruation^25^. We have recently shown that MenSC-exosomes act as potent inhibitors of tumor-induced angiogenesis in a xenograft prostate tumor model and also have anti-angiogenic effects on the breast cancer cell secretome^15^. On the other hand, no effect was observed on pancreatic cancer cell lines^15^. The fact that MenSC-exosomes show diverse effects on specific tumor types underscores the importance of studying the different cancer cell types to determine the scope of possible exosome-based treatments. Furthermore, the direct effect of MenSC-exosomes on endothelial cells and their secretome has not yet been described.

Exosome production involves three sequential steps: Cell culture with exosome-free media; collection of the conditioned medium, which contains secreted exosomes, and purification of the exosomes using different centrifugation protocols^26^. In current laboratory practice, adherent cells are mostly grown in 2D culture on plastic dishes or flasks. However, from this manufacturing process a limited quantity of exosomes is obtained - a fact that complicates translation of exosome treatments into the clinic. There has been much research on up-scaling to address this issue, especially at the level of cell culture systems, using successfully technologies such as microcarriers in stirred bioreactors and hollow-fiber bioreactors^27,28^. However, the shift from conventional bench-scale cell culture to large-scale culture platforms might alter the cellular phenotype or the metabolic status and consequently cause changes in the composition and function of exosomes. Thus, it is critical to evaluate whether the exosome product obtained from each manufacturing process maintains physical and proteomic characteristics as defined by the International Society of Extracellular Vesicles (ISEV)^29^ as well as their bioactive properties in target cells^28^.

In this work, we developed a platform of scalable exosome production using fiber-based microcarriers called BioNOC II and compared the yield to traditional dish cell culture. Subsequently, we studied the biological effect of the obtained MenSC-exosomes on endothelial cells *in vitro* and assessed the effect of exosome treatment on angiogenesis and tumor growth *in vivo* using the hamster buccal pouch (HBP) carcinoma model - a preclinical model that closely mimics the human OSCC^30^. This work provides relevant information about an anti-angiogenic therapy based on MenSC-exosomes and demonstrates for the first time the cytotoxic effect they exert on endothelial cells *in vitro* as well as a reduction of the tumor vasculature and tumor growth *in vivo*.

## Materials and Methods

MenSC characterization, exosome Purification and Characterization, Cytotoxicity, ELISA, tube formation and gene expression analysis are detailed in the supplementary data.

This work was revised and approved by the Ethics Committee of the Universidad de los Andes.

All animal studies were revised and approved by the Ethic Committee of Facultad de Medicina Clinica Alemana-Universidad del Desarrollo (Chile).

All experiments were carried out according to international standards and guidelines.

MenSC and UCMSC were obtained and used with the written and informed consent of donors following the institutional guidelines of Universidad de los Andes.

### Cell Culture

MenSC and umbilical cord (UC) MSC were isolated from menstrual fluid of the umbilical cord of three different healthy donors each and characterized according to ISCT^31^ criteria as we have previously described^15,22,32^. Human dermal fibroblasts were purchased from Lonza (cat. CC-2511, Lonza, Walkersville, MD USA). Human umbilical cord vein endothelial cells (HUVEC) and human microvascular endothelial cell line (HMEC-1) were obtained from the American Type Culture Collection (ATCC, Manassas, VA, USA). HUVEC and fibroblasts were cultured in Dulbecco’s Modified Eagle’s Medium (DMEM) (15-018-CV, Corning, New York, USA) supplemented with 10% fetal bovine serum (FBS) (Lonza, Walkersville, MD USA), 1% Penicillin/Streptomycin (P/S) (Life Technologies, Carlsbad, CA, USA) and 1 mM L-glutamine (Life Technologies). HMEC-1 cells were cultured in endothelial cells growth media 2 (EGM-2) (cc4147, Lonza). All cells were regularly tested for mycoplasma contamination using a mycoplasma detection kit (G238, Applied Biological Materials Inc., Richmond, BC, Canada). All cells were cultured in humidified incubation chambers at 37 °C with 5% CO_2_.

### Exosome Production

For exosome production, MenSC, UCMSC and fibroblasts between passage 4 and 8 were expanded in traditional 2D adherent cell culture and then seeded on BioNOC II micro carriers (CESCO Bioengineering, Taichung, Taiwan). For seeding, 8 × 10^6^ cells resuspended in 50 milliliters (ml) of DMEM with 10% FBS, 1% L-glutamine and 1% P/S and layered on top of two grams (g) of sterilized BioNOC II in a filter cap 250 ml Erlenmeyer flask (CLS431144, Sigma-Aldrich, Missouri, USA). The cells were maintained in static incubation during the first 16 hours (h) and were then supplemented with additional 200 ml of medium. For further culturing, the cells were maintained under constant agitation at 20 rounds per minute (rpm) on a rocking shaker (SK-R1807-E, DLAB Scientific, Beijing, China) in a humidified incubation chamber at 37 °C with 5% CO_2_. To assess cell confluence, five micro carriers were removed each day, washed with phosphate buffered saline (PBS) three times and the cell nuclei were stained with Hoechst solution 1:2,000 for 10 minutes (min) (63493, Sigma Aldrich). When the cells reached 70% confluence on the carriers, they were washed three times with 250 ml of PBS and their medium was changed to FBS-free, phenol red-free DMEM (17–205-CV, Corning) containing 1 mM L-glutamine and 1% P/S. After 72 h the supernatants were collected for exosome purification.

### Exosome Treatment

For exosome treatment, HUVEC or HMEC-1 cells were seeded at 2,500 and 5,000 cells/well in 96-well plates (using 100 µl medium), 10,000 and 20,000 cells/well in 24-well plates (using 300 µl medium) and 100,000 and 200,000 cells/well in 6-well plates (using 600 µl medium), respectively. After 48 h their growth medium was changed to DMEM with 1% P/S and 1% L-glutamine for HUVEC and endothelial basal medium (EBM) (CC-3156, Lonza) for HMEC-1. MenSC-exosomes were added at different concentrations to cell cultures for periods of 2–24 h depending on each cell type and experimental condition. Controls were performed with PBS vehicle instead of exosomes. Unless stated otherwise, experiments were repeated three times. Unless explicitly stated otherwise, MenSC-exosomes were used and referred to as exosomes or exos.

### Exosome Internalization

One hundred micrograms (µg) of pure exosomes were labeled with total protein stain CFSE (C34554, Thermo Fisher, Waltham, MA, USA) in 1 ml PBS according to the manufacturer’s instructions, washed by ultracentrifugation at 100,000 G for 3 h and resuspended in PBS. Three hundred µg of pure exosomes were labeled with DiR (60017, Biotium) at 71 µM for 1 h at 37 °C and, after incubation, unincorporated dye was removed by gel filtration using Exosome Spin Columns (MW 3000) (4484449, Invitrogen, Carlsbad, CA, USA). As dye controls, the CFSE and DiR were mixed with PBS instead of exosomes and washed as described above. The labeled exosomes were quantified using nano particle tracking analysis (NTA). HUVEC and HMEC-1 cells were seeded at a density of 10,000 cells/well in a 24-well format. After 24 h, the incubations of 4, 8 and 16 h were initiated with 38 × 10^3^ stained part/cell, labeled with CFSE for HUVEC and with DiR for HMEC-1 and in parallel with the corresponding dye control for 16 h. To quantitatively measure exosome uptake, the cells were trypsinized, washed with PBS and analyzed for CFSE or DiR signal using flow cytometry (FACS Canto II, Becton Dickinson (BD), Franklin Lakes, USA). For confocal microscopy images, HUVEC and HMEC-1 cells were seeded on top of a 10 mm cover glass coated with Poly-L-Lysine in a 4-well plate at 20,000 and 40,000 cells per well respectively. Cells were incubated with 20–50 × 10^3^ part/cell DiR stained exosomes and after 4, 8 and 16 h of incubation, the cells were washed three times with PBS and then fixed for 30 min at room temperature (RT) with 4% paraformaldehyde (PFA). Nuclei were stained with Hoechst 33342 (63493, Sigma Aldrich) 1:2,000 for 15 min at RT and preparations were mounted on a microscopy slide with fluorescence mounting medium (S3023, Dako, Santa Clara, CA, USA). Images were analyzed with confocal microscopy (SP8 AT CIAN, Leica, Wetzlar, Germany).

### Apoptosis Assays

Apoptosis was determined by flow cytometry using Annexin V and 7-AAD stain. Cells were seeded in 24-well plates 48 h before the experiment. For exosome treatment, cells were incubated with 20 µg/ml (HUVEC) or 40 µg/ml (HMEC-1) exosomes in basal medium. After 8 h, cell supernatants were mixed with the trypsinized cells in order to include detached dead cells in the analysis and cells were stained with Annexin V-FITC (556419, BD, Franklin Lakes, USA) and 7-aminoactinomycin D (7-AAD) (420403, Biolegend, San Diego, CA, USA) in Annexin V binding buffer (422201, Biolegend, San Diego, CA, USA). Cells were analyzed via flow cytometry (FACS Canto II, Becton Dickinson) in FITC and PerCP5.5 and gates for quantification were set using single stain tubes. Flow cytometry data was analyzed using FlowJo v.10 (Ashland, Oregon, USA).

### In Vivo

Oral squamous cell carcinoma (OSCC) was induced in 16 female 8 weeks old Syrian golden hamsters (*Mesocricetus aureatus*) by applying a N°4 camel-hair brush soaked with 50 µl of mineral oil (Sigma-Aldrich) or 50 µl of 0.5% 7,12-Dimethylbenzanthracene (DMBA) (Sigma-Aldrich) dissolved in mineral oil three times a week into their right buccal pouch^33–35^. Animals were housed at 22 ± 2 °C, with a 12:12 h light-dark cycle, water and food *ad libitum*, and body weight of the hamsters was measured twice a week. Twelve weeks after the initiation of OSCC induction, hamsters were deeply anesthetized by inhalation of Sevoflurane (Abbot, Tokyo, Japan). Buccal pouches were uncovered and tumors were measured with a digital caliper and photographed using a digital camera (FUJIFILM-Finepi HS20 EXR). The lesion volume was calculated using the formula: tumor volume (mm^3^) = 0.52 × [width(mm)]^2^ × length (mm) and the tumor increase was estimated as the ratio between final and initial volumes^36^. After 16 weeks of exposure to DMBA, the tumors reached the carcinoma stage and the DMBA treatment was suspended. For exosome treatment, animals were randomly distributed to experimental groups when lesions reached the stage of carcinoma^33,35^. The animals were anesthetized, their buccal pouch exposed using forceps and MenSC-exosomes (20 µg in 50 µl PBS) or PBS (vehicle) were injected into the base of the tumor, twice a week for 2 weeks using a 30-gauge needle (Becton Dickinson). Two independent *in vivo* experiments were carried out.

### Histopathology of Tumors

Four weeks after vehicle or exosome administration, hamsters were euthanized by an intraperitoneal injection of Xylazine and Ketamine. Buccal pouches were harvested and tumors were resected, weighted, and sagittally dissected into two parts, one of which was immediately frozen in liquid nitrogen and stored at −80 °C for gene expression studies, while the other was stored in 4% paraformaldehyde for histopathological analysis. Tissue sections of 4 µm were stained with hematoxylin and eosin (H&E) (Merck, Darmstadt, Germany) and visualized with a light microscope (DM2000, Leica). Two independent pathologists performed histological analysis in blind.

### Fluorescein Angiography

Hamsters were injected intracardially with 400 µl of PBS containing 20 mg of 2 × 10^6^ molecular weight fluorescein-dextran (Sigma-Aldrich). Five minutes later, they were euthanized and tumors were fixed in 4% paraformaldehyde. Four-micrometer tumor slices were observed and photographed with the Fluoview FV10i confocal microscope (Olympus) and six random fields per animal were analyzed. The fluorescence area, representative of vascular lumen area, was quantified using the ImageJ 1.34 software, NIH^37,38^. To confirm the vascular lumen, labeled by fluorescein-dextran, tissue sections of 4 µm were deparaffinized, rehydrated, blocked with 5% of FBS and incubated overnight at 4 °C with a dilution of anti-VE-cadherin (sc-6458 Santa Cruz, Santa Cruz, CA, USA). Then samples were washed and incubated for 2 h at RT with the secondary antibody (Alexa555). Cross-reactivity of the secondary antibody was tested by incubating samples without the primary antibody. Nuclei were counterstained with 4′6′-diamidino-2-phenylindole (DAPI) (Sigma-Aldrich). Samples were analyzed by confocal microscopy.

### Statistical Analysis

To compare two conditions, unpaired two-tailed student’s t-test were used. Statistical analyses were carried out using GraphPad Prism 5 (Graphpad Software, Inc. San Diego, CA, USA). Statistical significance was shown as *p < 0.05; **p < 0.01; ***p < 0.001. Error bars represent the standard error of the mean (SEM).

## Results

### Micro carrier-based cell culture is an efficient way to produce exosomes and does not alter stem cell characteristics

Since exosome production is a time-consuming process, we aimed at improving the standard protocol for exosome production^15,26,39,40^ in terms of yield and hands-on cell culture time. We cultured MenSC on micro-carriers (BioNOC) for exosome production. Firstly, we examined whether this distinct culture format would affect the MenSC stem cell characteristics. Supplementary Fig S1A shows the histograms for positive MSC markers (CD73, CD90 and CD105) and negative markers (HLA-DR, CD14, CD19, CD34 and CD45) and a summary of percentage positive cells in table format comparing dish culture and carrier culture. Supplementary Fig S1B shows that MenSCs that were previously cultured on carriers or on traditional cell culture dishes differentiated equally well into adipocytes, chondrocytes and osteocytes. There was no difference in marker expression between the two culture systems and both were compliant with the ISCT guidelines^31^ for MSC characterization, showing that growth in BioNOC carriers did neither compromise the MenSC immunophenotype nor their tri-lineage differentiation potential. Therefore, we conclude that MenSCs maintain their characteristics in BioNOC-based cell culture.

**Figure 1:**
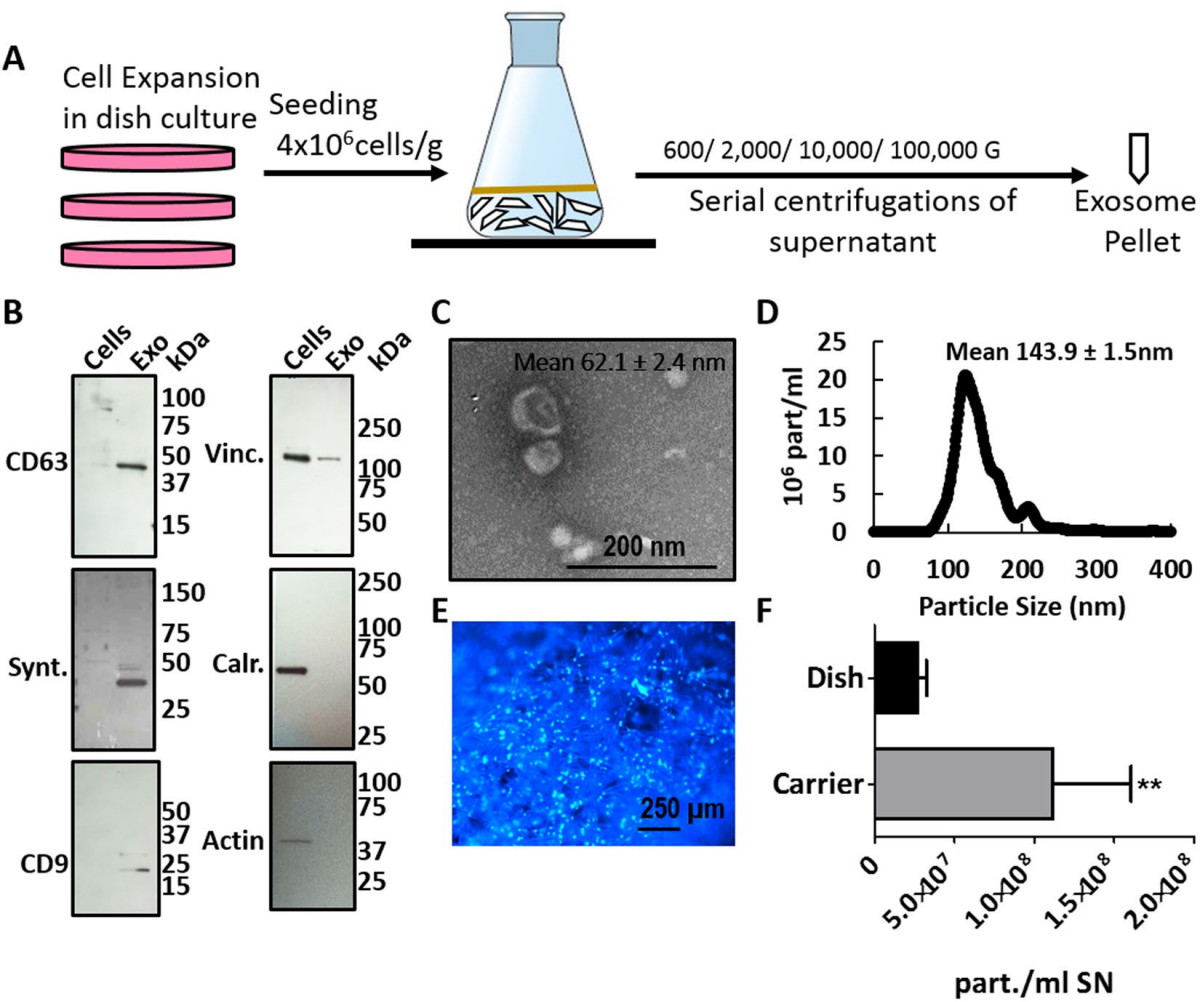
Carrier-based cell culture increases exosome yield. (**A**) Schematic overview of exosome production process. MenSC were expanded on 2D cell culture dishes and then seeded on BioNOC II. After 72 h in DMEM with 10% FBS, the medium was changed to serum-free DMEM for 72 h for exosome production. For purification, the supernatant was collected and processed in serial centrifugations. (**B**) Western Blot with 15 µg of cell lysate (Cells) and exosomes (Exo). To confirm the purity of the exosomes, positive exosomal markers CD63, Syntenin and CD9 and negative exosomal markers Vinculin (Vinc.) and Calreticulin (Calr.) and β-Actin (actin) were analyzed. (**C**) Scanning electron micrograph of purified exosomes, magnification 60,000x. (**D**) Size distribution of exosomes determined by nanosight showing that the highest abundance of particles was below 200 nm. (**E**) Hoechst-stained MenSC on BioNOC II carrier, showing a typical confluence for exosome production. (**F**) Yield of purified exosomes in PBS as Particles (part)/ml of initial cell culture supernatant (SN) are shown for 2D (N = 32) and 3D culture (N = 10). Analysis: unpaired t-test. Bar graphs show average values, error bars: SEM.

We then proceeded to characterize the exosomes that were secreted by BioNOC-cultured MenSCs. Figure 1A shows a schematic overview of the MenSC culture on the fiber-based micro carriers BioNOC II that was employed for exosome production. The exosome purification protocol was maintained as standard protocol using ultra centrifugation^15,26,39,40^. The enrichment of the exosomal markers CD63, Syntenin and CD9 as compared to cell lysate and the absence of cytoskeletal markers β-Actin and Vinculin and ER marker Calreticulin was confirmed via western blot (Fig. 1B, Supplementary Fig S2). CD63 and CD9 are tetraspanins enriched in endosomes where exosomes originate and Syntenin is a key regulator of exosome biogenesis. The exosomes produced from carrier-based culture maintained their characteristic morphology and had an average size of 62.1 ± 2.4 nm when analyzed by electron microscopy and 143.9 ± 1.5 nm when analyzed with NTA as shown in Fig. 1C and D, respectively. For confluence determination, micro carriers were stained with fluorescent DNA stain Hoechst as shown in Fig. 1E. As shown in Fig. 1F, the carrier-based BioNOC II culture produces a significantly higher number of particles per ml of supernatant compared to dish culture. Since supernatant volume is a relevant measure and limitation for subsequent purification steps in the ultra-centrifuge, we normalized the particle number to initial supernatant volume. On average, 1.2 × 10^8^ ± 0.56 × 10^8^ exosomal particles were produced per ml of supernatant in carrier-based culture, whereas traditional dish culture produced only 0.27 × 10^8^ ± 0.06 × 10^8^ particles per ml supernatant. Furthermore, the cell culture hands-on time for producing the same number of exosomes could be reduced dramatically since processing large numbers of cells in dish culture is very time-consuming. In conclusion, BioNOC II-based culture of MenSC is a more time-efficient alternative for exosome production that does not alter the general exosomal characteristics.

### Exosomes are internalized by endothelial cells

**Figure 2:**
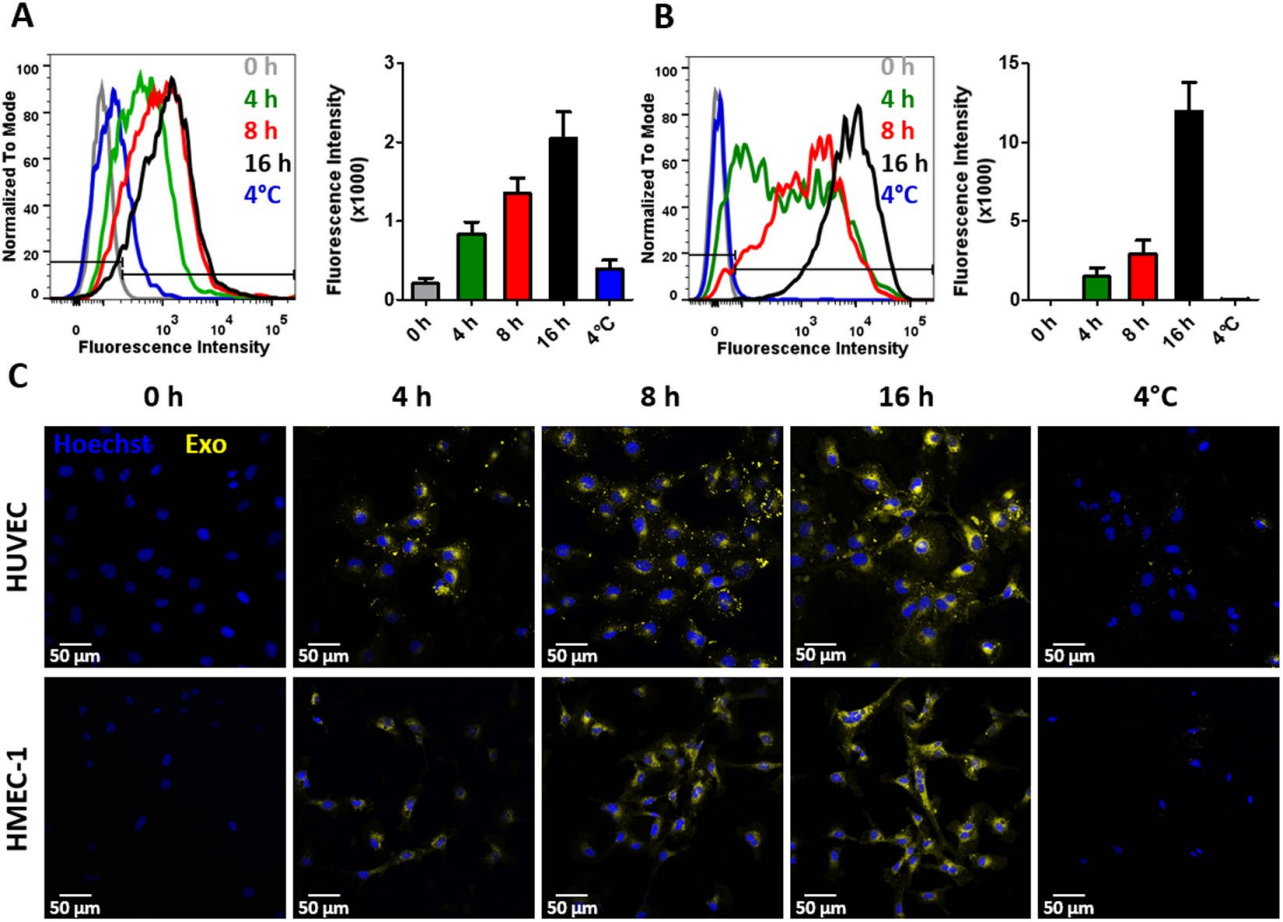
MenSC-exosomes rapidly enter endothelial cells in culture. (**A**,**B**) Representative histograms and quantification of exosome uptake analyzed via FACS for HUVEC (**A**) and HMEC-1 (**B**) at different time points. (**C**) HUVEC and HMEC-1 were incubated with DiR labeled exosomes and analyzed by confocal microscopy. Scale bar: 50 µm. Bar graphs show average values, error bars: SEM. N = 3.

We have previously shown that MenSC-exosomes are internalized by tumor cells and modulate their angiogenic secretome^15^. To determine whether MenSC-exosomes would also have a direct effect on endothelial cells, we first assessed their internalization in two different types of endothelial cells: HUVEC and HMEC-1. Internalization was quantified in a time-dependent fashion detecting stained exosomes within endothelial cells via FACS and confocal microscopy. As shown in Fig. 2A and B, exosomes were efficiently taken up by HUVEC and HMEC-1 cells on increasing amounts over the course of 16 h. The mean fluorescence intensity (MFI, geometric mean) of HUVEC increased from 832 ± 154 at 4 h to 1349 ± 196 after 8 h and reached 2046 ± 336 after 16 h as more stained exosomes were internalized (CFSE-stained). The negative control at 4 °C had a MIF of 392 ± 110. The MIF of HMEC-1 was 1472 ± 556, 2908 ± 870, 11943 ± 1843 at 4, 8 and 16 h, respectively and 14 ± 1 at 4 °C as they internalized stained exosomes (DiR-stained). The low MIF at 4 °C confirms an active internalization process of the exosomes into endothelial cells. A dye-only control was negative (data not shown) indicating that our washing steps successfully removed all free dye, such that any observed fluorescence signal stemmed from stained exosomes. To determine whether exosomes are actually internalized or only membrane-bound, we confirmed their cytoplasmic localization via confocal microscopy as shown in Fig. 2C. Exosomes for confocal microscopy were stained with DiR, shown in yellow. The confocal microscopy confirmed effective and time-dependent internalization of exosomes into HUVEC and HMEC-1.

### Exosomes induce apoptosis in endothelial cells

**Figure 3:**
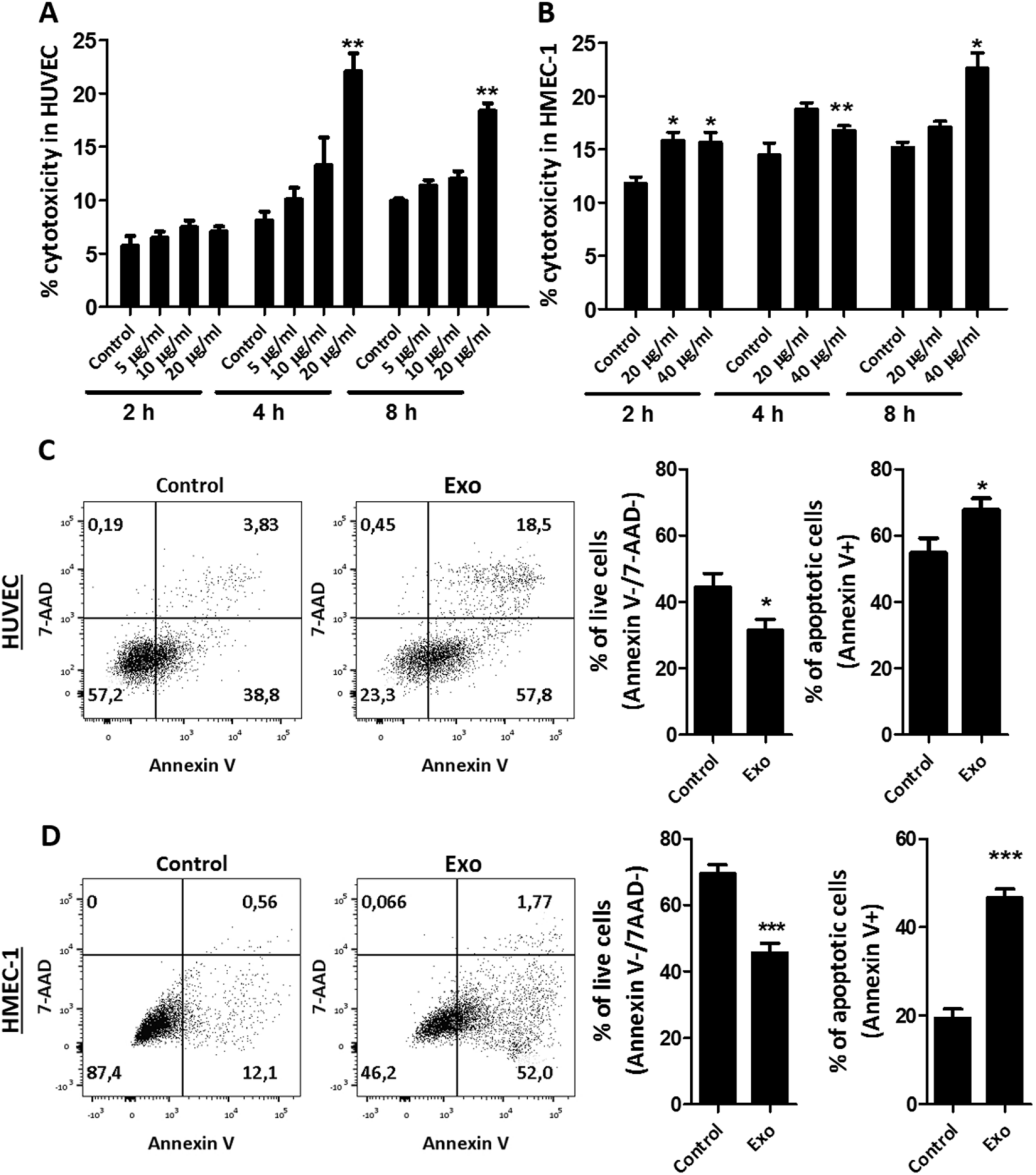
Exosomes induce cell death in endothelial cells. (**A**,**B**) Percentage cytotoxicity determined by LDH activity in HUVEC (**A**) and HMEC-1 (**B**) supernatant after 2, 4 and 8 h using 20 µg/ml and 40 µg/ml exosome treatment, respectively. (**C**) Annexin V/7-AAD viability stain analyzed HUVEC cells after 8 h of exosome treatment (20 µg/ml). (**D**) Annexin V/7-AAD viability for HMEC-1 cells after 8 h of exosome treatment (40 µg/ml), viable cells correspond to lower bottom quadrant and apoptotic cells to top and bottom right quadrant. Analysis: unpaired t-test. Bar graphs show average values, error bars: SEM.

As endothelial cells are responsible for angiogenesis, we wondered whether the anti-angiogenic effect of exosomes we have previously described^15^ would be related to direct cytotoxic effect on endothelial cells. To assess cytotoxicity, lactate dehydrogenase (LDH) activity in supernatants was measured. Indeed, we found that exosome treatment, even at short time-points, induced cytotoxicity in a dose-dependent manner in both endothelial cell types. In HUVEC, a significant increase in cytotoxicity was firstly achieved after 4 h of incubation with 20 µg/ml of exosomes, where the percentage of cytotoxicity was 8.1 ± 0.88% in control and 22.1 ± 1.67% after exosome treatment. After 8 h incubation, control values were at 9.9 ± 0.21% and exosome-treated cells at 18.4 ± 0.72% cytotoxicity (Fig. 3A). In the case of HMEC-1, 20 µg/ml were not sufficient to evoke a significant increase in cytotoxicity except after 2 h. Therefore, the dose was increased to 40 µg/ml for this cell line which lead to a significant increase in cytotoxicity after 2, 4 and 8 h of incubation. The percentage cytotoxicity was 10.8 ± 0.66%, 11.6 ± 0.68%, 14.4 ± 0.83% in control and 15.6 ± 0.95%, 16.8 ± 0.45% and 22.6 ± 1.46% in exosome-treated HMEC-1 after 2, 4 and 8 h, respectively, as shown in Fig. 3B. Since LDH may also be present in exosomes, we tested they basal level of LDH activity in a full dose of MenSC-exosomes in DMEM and EBM and found that there was no detectable LDH activity originating from the exosomes (data not shown), demonstrating that the measured LDH stemmed from the endothelial cells. Due to the short half-life of free LDH, we did not assess longer time-points in the cytotoxicity assay as they would not represent an accumulative cytotoxicity of the whole incubation period. Cells were also analyzed with Annexin V and 7-aminoactinomycin D (7-AAD) stain which are used to label early and late apoptosis, respectively. Annexin V binds to phosphatidylserine which is exposed during apoptosis and 7-AAD labels cells with damaged membranes. We found that the percentage of viable cells decreased significantly after 8 h exosome treatment in HUVEC (Fig. 3C). The viability of control cells was 44.4 ± 4.2% and exosome- treated cells 31.5 ± 3.3% and apoptotic cells (Annexin V^+^ cells) comprised 54.8 ± 4.4% in control and 67.8 ± 3.4% in treated HUVEC. Although the viability of HUVEC cells measured using Annexin V/7AAD staining was relatively low, this is most likely due to the process of cell detachment and post-detachment washing steps as the cell’s morphology in cell culture was not visibly altered and a non-starving control showed the same values (data not shown). Also in HMEC-1 cells, the cytotoxic effect of MenSC-exosomes could be confirmed using 40 µg/ml in the Annexin V/7-AAD assay (Fig. 3D). The percentage of viable cells was significantly decreased from 69.4 ± 2.8% in control to 45.6 ± 2.9% in exosome-treated HMEC-1. Apoptotic cells increased significantly with exosome-treatment as they comprised 19.5 ± 2.0% in control and 46.6 ± 2.0% in treated HMEC-1. These data indicate that exosomes have cytotoxic effects that induce cell death in endothelial cells.

### Exosomes affect angiogenic activity of endothelial cells by modulating VEGF secretion

**Figure 4:**
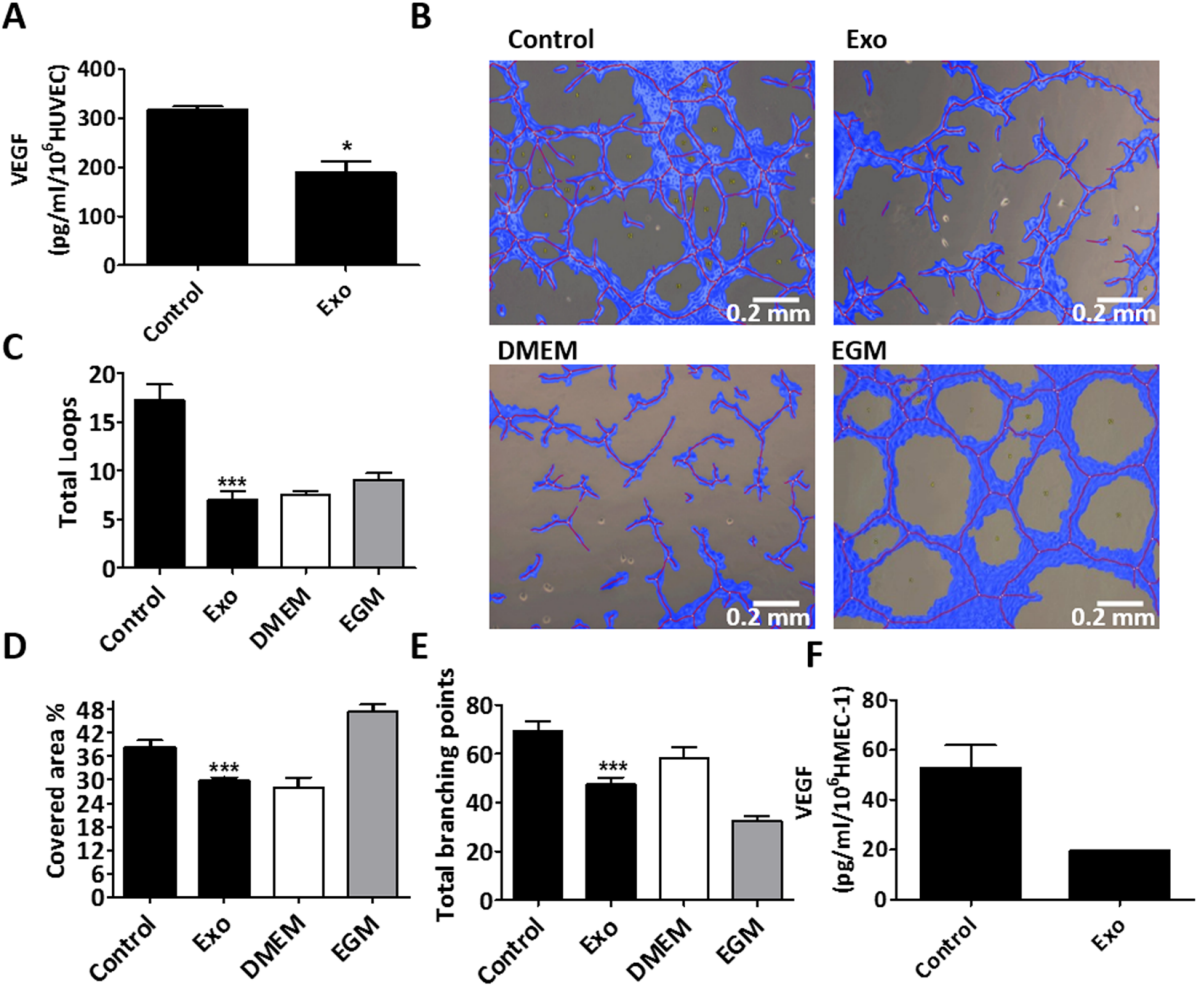
Inhibition of angiogenesis *in vitro* by exosome treatment. (**A**) VEGF secretion by HUVEC after 24 h treatment with 20 µg/ml MenSC-exosomes. (**B**) Representative photographs of HUVEC tube formation on matrigel *in vitro*, using 24 h HUVEC supernatant without (control) and with 20 µg/ml exosomes, magnification 10x. The negative and positive controls were DMEM and EGM, respectively. (**C**) Total loops. (**D**) Percentage covered area. (**E**) Total branching points. (**F**) VEGF reduction in HMEC-1 after 24 h treatment with 40 µg/ml MenSC- exosomes. Analysis: unpaired t-test. Bar graphs show average values, error bars: SEM.

To test whether MenSC-exosomes could directly modulate the angiogenic potential of endothelial cells, we incubated HUVEC and HMEC-1 cells with exosomes for 8 and 24 h. Firstly, we determined VEGF secretion levels since this protein is the most relevant factor for angiogenesis^41^. Figure 4A shows that exosome-treatment significantly reduced VEGF secretion of HUVEC after 24 h of incubation from 318.0 ± 6.0 to 190.0 ± 22.0 pg/ml/1 × 10^6^ cells. Interestingly, 8 h were not sufficient to evoke a significant reduction of VEGF levels (Supplementary Fig S3). The same figure shows a dose-response pattern when incubating HUVEC with 5, 10 and 20 µg/ml exosomes. Since exosomes reduced VEGF levels, we asked whether the endothelial cells’ overall angiogenic potential would equally be reduced by exosome treatment. Therefore, we carried out *in vitro* tube-formation assays on matrigel using conditioned medium of HUVEC treated with exosomes for 8 and 24 h. In accordance with VEGF levels, we observed that conditioned medium from 8 h did not affect angiogenesis (Supplementary Fig S4) but 24 h exosome-treated endothelial cells induced less tube formation than control cells (Fig. 4B). Figure 4C–E show that after 24 h exosome incubation, there was a significant decrease in total loops from 17.27 ± 1.63 to 7.0 ± 0.91, percentage of the covered area from 38.26 ± 1.82 to 29.72 ± 0.87, total branching points from 69.27 ± 3.8 to 47.50 ± 2.7 and total tube length from 20940 ± 681.7 to 16890 ± 797.6 (Supplementary Fig S5) in exosome-treated HUVEC cells with respect to control HUVEC conditioned medium. DMEM was used as a negative technical control since it was the basal medium of the conditioned media and EGM was used as a positive technical control as it contains angiogenesis-promoting factors such as VEGF. In the case of HMEC-1, 40 µg/ml of exosomes induced an important reduction of VEGF levels that was, however, not significant (p = 0.0686 (Fig. 4F). Since HMEC-1 need to be grown in growth factor-enriched medium but experiments were performed in basal medium, the HMEC-1 conditioned medium was not able to induce tube-formation on HMEC-1-cells. In order to test whether the observed anti-angiogenic effect was MenSC-specific, we examined the effect of UCMSC-exosomes and dermal fibroblast exosomes on HUVEC cells as shown in supplementary Fig S6. Interestingly, these exosomes showed opposite effects with respect to MenSC-exosomes. UCMSC-exosomes induced a higher percentage of covered area of 61.23 ± 4.146 compared to the control with a percentage of 43.61 ± 2.611, a higher number of total loops with 26.67 ± 2.279 as compared to 14.22 ± 1.245 in the control and a larger total tube length of 24550 ± 953.3 pixels as compared to 20620 ± 696.9 in the control. Similarly, fibroblast exosomes induced a higher percentage of covered area, more total loops and a longer total tube length (72.50 ± 3.113%, 41.00 ± 5.132 and 27880 ± 1616 pixels, respectively). Taken together, these data suggest that the anti-angiogenic effect of MenSC-exosomes is a unique feature of this type of exosomes and not necessarily attributable to other cell types.

### Exosomes inhibit tumor growth in vivo

**Figure 5:**
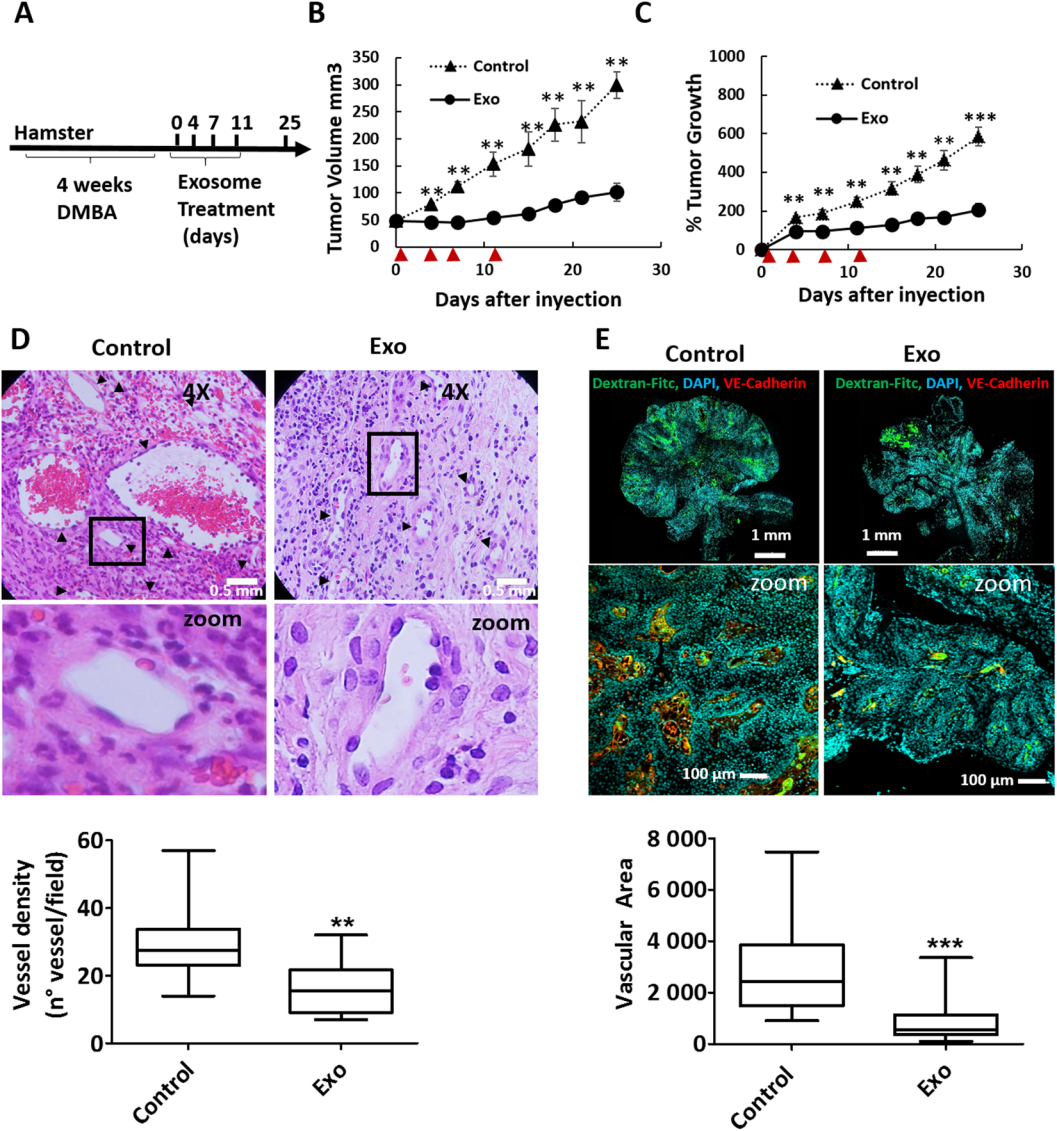
Tumor growth and angiogenesis is significantly reduced by exosome treatment. (**A**) Scheme of experimental design. Tumors were induced with four weeks of DMBA treatment and four injections of exosomes were administered every 3–4 days (**B**,**C**) Tumor growth in mm_3_ tumor volume and relative tumor growth.  indicates days of exosome treatment. Control tumors ar e shown as triangles and exosome treated tumors as circles. N = 9 (control), N = 8 (Exo). (**D**) Histological sections of tumors at day 25 (end-point) with Hematoxylin and eosin stain (H&E). Quantification of vessel density based on H&E sections is shown below. ▲ indicates vessels. (**E**) Dextran-Fitc (green), VE Cadherin (red) and Hoechst (blue) stained histological sections of tumors at day 25 (end-point). Upper panel: complete tumors, lower panel: zoom of selected tumor area. VE-Cadherin is only shown for zoom (lower panel). Graph shows quantification of vascular area based on dextran stain. Analysis: unpaired t-test. Bar graphs show average values, error bars: SEM.

Squamous cell carcinoma of the buccal pouch in the Syrian hamster closely models oral squamous cell carcinoma in humans as it has similar aberrant gene expression patterns^30^. We therefore argued that the anti-angiogenic effect of MenSC-exosomes might present advantages for the treatment of this tumor type. Four exosome injections (20 µg each) were carried out after the tumor reached a size of 50 mm^3^, as schematized in Fig. 5A. The exosome treatment could significantly inhibit tumor growth when compared to vehicle treated controls, even after only one injection, as early as day four (Fig. 5B,C). At end-point, 25 days after treatment initiation, the tumor volume was smaller in exosome treated tumors (101.4 ± 19.8 mm^3^) than in control tumors (354.0 ± 59.5 mm^3^). The same was observed in percentage of growth that was 206.2 ± 40.1% in exosome treated tumors with respect to baseline and 585.2 ± 40.8% in control tumors. Exosome treated tumors maintained their size with a tumor volume of 48.39 ± 2.8 mm^3^, 46.09 ± 3.6 mm^3^, 45.85 ± 12.2 mm^3^ and 54.54 ± 7.8 mm^3^ at days 0, 4, 7 and 12, respectively. Control tumors, on the other hand, grew steadily from 45.33 ± 20.0 mm^3^ to 79.11 ± 8.1 mm^3^, 92.015 ± 11.7 mm^3^ and 144.40 ± 24.8 mm^3^ at days 0, 4, 7 and 12, respectively. The average tumor weight at day 25 was significantly lower in treated tumors as compared to control (95 ± 23 mg and 237 ± 38 mg, respectively) and the hamsters’ body weight was not affected by the treatment which suggests that it had no harmful effects on the animals (Supplementary Fig S7). Since it had been observed that exosome treatment reduced angiogenesis *in vitro*, we analyzed tumor vasculature of vehicle and exosome-treated tumors at the experimental end-point. We found that both vessel density and vascular area were significantly reduced by exosome-treatment (Fig. 5D,E). The vessel density, quantified based on H&E stained histological sections, was reduced from 29.9 ± 3.1 in control to 16.3 ± 2.3 vessels/field in exosome treated tumors and the vascular area, quantified based on Dextran-Fitc stained histological sections, was reduced from 2842.3 ± 252.2 to 840.3 ± 117.9 pixels. We also analyzed mRNA levels of Ki67, p53 and VEGF in tumors at the experimental end-point (day 25) but found no significant difference between exosome-treated and control tumors (Supplementary Fig S8). The fold change of VEGF expression compared to the average of control tumors was on average 0.92 ± 0.30. In the case of p53, the fold change was 1.08 ± 0.34 and in the case of Ki67 0.73 ± 0.23.

## Discussion

It is well-known that the therapeutic effects of stem cell therapy are in large proportion caused by their secreted factors. In the last years, these paracrine mechanisms have been linked to exosomes^42,43^, membrane-enclosed nanovesicles that shuttle biomolecular cargoes that can effectively alter the biological properties of target cells^44^. Since we expect that the number of clinical trials using exosome treatments will rapidly increase in the coming years, there is a need to develop a robust manufacturing technology that ensures enough exosomes to treat a significant number of patients. Along with this, it is critical to guarantee that the exosome-based product meets the requirements for its identification to control the manufacturing quality^29,44^. With this in mind, we presented in this report an alternative way for the up-scaling exosome production using the fiber-based micro-carriers BioNOC II. We showed that the micro-carrier based cell culture did not affect stem cell markers or differentiation potential of MenSCs, indicating that it is a feasible culture method for MSCs. Exosomes isolated using this methodology presented round-shaped morphology and size and biochemical markers according to the criteria suggested by the exosome community^29^. There are several advantages of BioNOC II carrier culture. First, exosome production costs can be reduced as compared to dish cell culture since less medium and plastic material is used. Also, BioNOC II micro-carriers can be easily purchased and implemented in a research laboratory since there is no need of pump systems or specific devices. It is therefore a convenient alternative to hollow-fiber bioreactors. Furthermore, hands-on time is significantly reduced in cell culture steps when compared to the large number of cell culture dishes which would be needed to bear the same number of cells. Finally, we showed that the particle yield per ml conditioned medium was superior to dish cell culture systems. Importantly, though, the control of environmental parameters during cell culture is key to avoid phenotypic and metabolic changes in cells that may result in an alteration of the exosomes cargoes^28^. In our present study, we could replicate the MenSC-exosome’s anti-angiogenic effect in tumors that we had obtained previously with exosomes from dish cell culture^15^. This suggests that the MenSC-exosomes were not altered by the change in culture method. Undoubtedly, to proceed towards the translational phases, it is also recommended to perform a functional characterization of exosomes defined by their content in proteins and nucleic acids. Knowing the active compounds in exosome-based products and their mechanism of action, will allow the implementation of effective quality control in the manufacture of exosome-based therapies and may reveal subtypes of exosomes with specific functions.

The uptake of the MenSC-exosomes by endothelial cells was an energy-dependent process. Each type of endothelial cells showed its own kinetics of exosomes internalization, confirming previous findings in other models that demonstrate that the mechanism of internalization is a specific process to each cell type^45–47^. The mechanism responsible for MenSC-exosome internalization was not studied in this work although it is an interesting question to resolve. From an oncological point of view, it would be important to study the exosome internalization in an acidic environment that mimics the tumor microenvironment since the pH is relevant in the regulation of the exosome uptake by cells^48^.

Functionally, we observed that exosome-treatment lead to cell death and reduced angiogenesis in endothelial cells. Previously, we have shown that MenSC-exosomes inhibited VEGF-secretion of tumor cells^15^ and here we could show that the same effect is obtained when directly incubating endothelial cells with exosomes. Therefore, the anti-angiogenic effects of MenSC-exosomes observed *in vivo* are likely due to a combination of effect on both, tumor and endothelial cells. One limitation of studying the endothelial cell line HMEC-1 is that they are dependent on media providing growth factors such as VEGF. This complicates longer incubation times with exosomes in basal medium. We further found that exosome treatment induced cell death and cytotoxicity in HUVEC and HMEC-1. Monocyte-derived exosomes have been shown to induce apoptosis in endothelial cells^49^ but to our knowledge this is the first report of such an effect from MSC-exosomes. An interesting question for future studies is whether a causal relationship exists between VEGF secretion and cytotoxicity in exosome-treated endothelial cells, addressing cytotoxic effect *in vivo*. We demonstrated in tube formation assays that the specific source of cells affects their exosomes’ function since UCMSC- and fibroblast exosomes showed pro-angiogenic properties. We have previously shown that exosomes derived from MSCs of bone marrow (BM), adipose tissue (AT), umbilical cord (UC) and menstrual fluid shared physical and biochemical characteristics^39^. However, their biological actions still differed^12,21^ due to varying composition of their molecular content^20^. Recently, we have demonstrated opposing effects for exosomes derived from BMSC or MenSC on tumor angiogenesis^15^. This suggests that observed biological effects of exosomes from one type of MSC should not be extrapolated to those secreted by other MSCs. Likewise, the anti-angiogenic effect of MenSC-exosomes differs for different tumor cell lines^15^ indicating once again the need to study the effects of one type of exosomes on one specific type of cancer. This complements our results in previous publications, namely that bone marrow mesenchymal stem cell (BMSC) exosomes are pro-angiogenic^15^ and results of others describing a pro-angiogenic effect of UCMSCs^50^.

With this in mind, we decided to test the effect of MenSC-exosomes on the vasculature of the most prevalent neoplasm of the oral cavity, the OSCC. The functional experiments in the HBP carcinoma model demonstrate that MenSC-exosomes also exert an important anti-tumor effect *in vivo*. Our findings show that the anti-tumor effect was associated with a loss of the tumor vasculature, observed through the reduction of the number and size of the blood vessels. Based on our *in vitro* results, we hypothesize that the smaller tumor size was caused by weaker angiogenesis which is known to be relevant for tumor growth. However, the possibility that an angiogenesis-independent mechanism slowed the tumor growth which in turn led to reduced angiogenesis cannot be ruled out completely. The difference in tumor growth was persistent until the end of the study (day 25) and the vessel density and number was also analyzed at this time-point. However, the VEGF expression analyzed by qPCR was not homogeneous at the experimental end-point in exosome-treated tumors and showed no overall difference with respect to control tumors. It is likely that MenSC-exosomes exert their anti-angiogenic effects not only via VEGF but also by VEGF-independent mechanisms which would explain why the treated tumors show diverse levels of VEGF mRNA despite having consistently smaller tumor volumes. Since exosomes contain several active compounds such as miRNAs and proteins, different miRNAs might cause effects in different signaling pathways and cell functions. On the other hand, the similar VEGF levels in control and exosome-treated tumors could also be attributed to the time-point of analysis as animals were only sacrificed two weeks after the last exosome administration and it is known that VEGF-expression can quickly return to normal or even higher levels after withdrawing anti-angiogenic therapies^51–53^. The anti-angiogenic effect of MenSC-exosomes was previously described by us in prostate and breast cancer^15^. Whether MenSC-exosomes exert their effect through the transfer of proteins, miRNAs or a combination of both is a key question to resolve in future investigations. As mentioned above, deciphering the active compound(s) will provide a better understanding of the mechanism of action of exosomes and pave the way to future enhancements of therapeutic effects while allowing better predictions of drug interactions, side effects and patient responder status. In line with our findings, other groups have also shown an antitumoral effect of MSC-exosomes. Lee and colleagues demonstrated that murine BMSC-exosomes suppress the tumor angiogenesis by down-regulating VEGF expression in a breast tumor model, partially mediated by the transfer of miRNA-16^16^. Del Fattore and colleagues have shown that microvesicles (MVs) secreted by BMSC and UCMSC inhibit the proliferation and induce apoptosis of glioblastoma cells^21^. Likewise, BMSC-microvesicles have been shown to be effective when inhibiting the growth of hepatic and ovarian cancer cells and Kaposi’s sarcoma both *in vitro* and *in vivo*^54^.

HBP is a suitable *in vivo* model to study human OSCC. HBP tumors share morphological and histological characteristics as well as expression patterns of many biochemical and molecular markers that are also expressed in human OSCC^30,55^. However, one limitation of the hamster model is the lack of commercially available reagents such as antibodies specific or cross-reactive to hamsters. This limits the extension of further research. In the future, it will be essential to test the effectivity of the exosomes in a model of metastatic disease. For this, it is necessary that exosomes localize to the tumor sites *in vivo* after systemic administration. It has already been demonstrated that exosomes secreted by different cell types are able to reach the tumor^56–61^. However, different types of exosomes could potentially show distinct innate homing abilities *in vivo*^61^. Therefore, it will be important to conduct studies specifically in MenSC-exosomes to evaluate their biodistribution and tumor homing properties.

## Supplementary information

Supplementary Data
