# Supplementary material for: Stem cell exosomes inhibit angiogenesis and tumor growth of oral squamous cell carcinoma
Source: Sci Rep. 2019 Jan 24;9:663. doi: 10.1038/s41598-018-36855-6 (PMC6345809; doi:10.1038/s41598-018-36855-6)
Supplement: Supplementary file 1 — Supplementary Data [file 41598_2018_36855_MOESM1_ESM.pdf]

Supplementary data

Stem cell exosomes inhibit angiogenesis and tumor growth of oral squamous cell carcinoma

Leonie Rosenberger[1*], Marcelo Ezquer[2*], Fernando Lillo-Vera[3,4], Paulina L. Pedraza[2], María Ignacia Ortúzar[1], Paz L. González[3], Aliosha I. Figueroa-Valdés[1,4], Jimena Cuenca[1,3,4], Fernando Ezquer[2], Maroun Khoury[1,3,4], Francisca Alcayaga-Miranda[3,4&]

* These authors have contributed equally to this work

& Corresponding author **falcayaga@uandes.cl**

Supplementary Figures

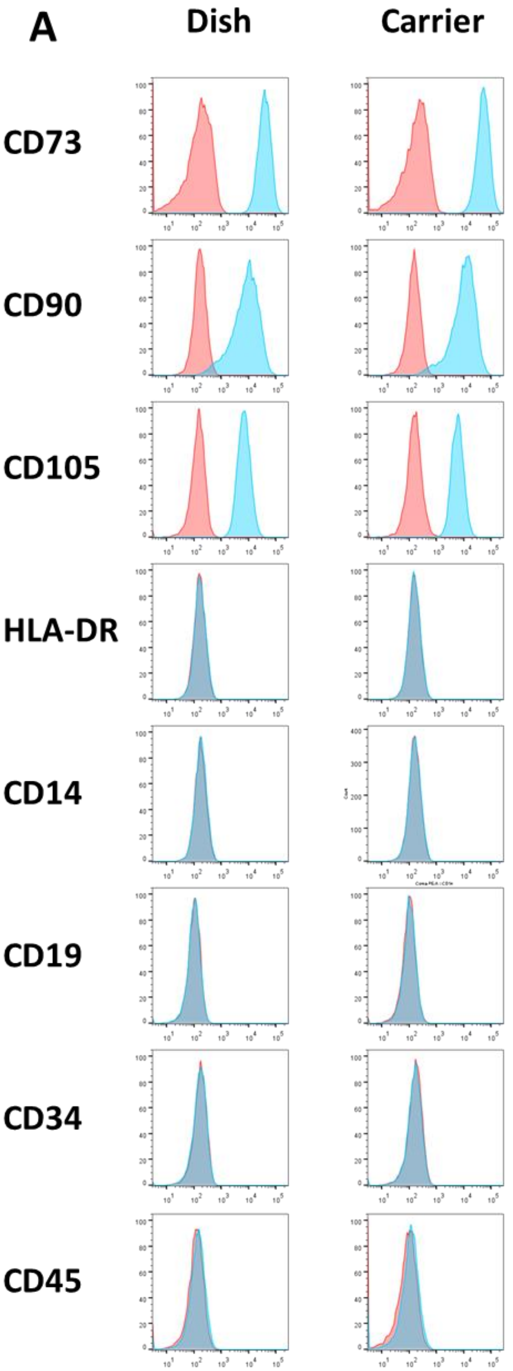

Marker	Dish Mean	Dish SE	Carrier Mean	Carrier SE
CD73	100	0,00	100	0,00
CD90	95	1,85	96	0,65
CD105	100	0,00	100	0,05
HLA-DR	0	0,01	0	0,00
CD14	0	0,02	0	0,03
CD19	0	0,00	0	0,01
CD34	0	0,01	0	0,00
CD45	0	0,02	0	0,02

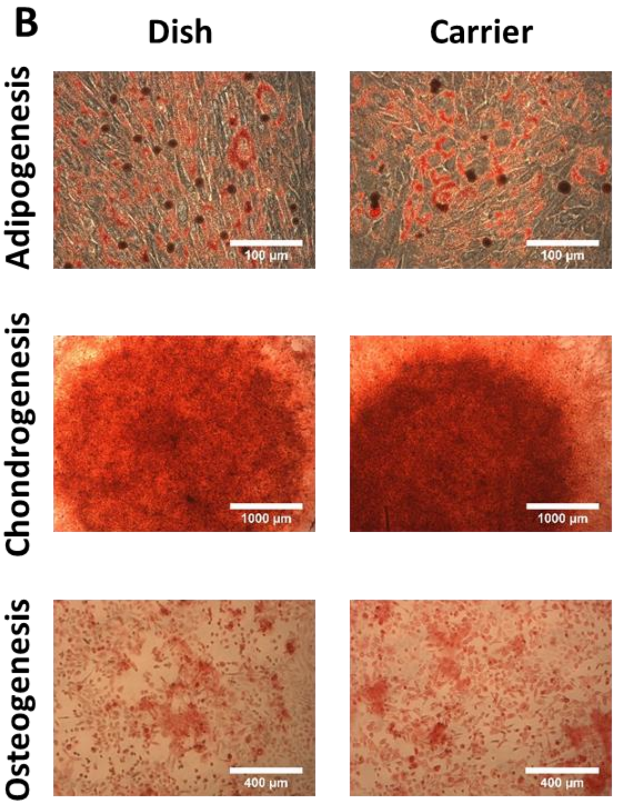

Figure S1: MenSCs stem cell characterization comparing dish- and carrier- based cell culture. (**A**) Flow cytometry histograms of FMO control (red) and MenSCs (blue) showing positive markers (CD73, CD90, CD105) and negative markers (HLA-DR, C14, CD19, CD34 and CD45). Table shows the surface marker profiles presented as percent positively stained cells. Data are presented as mean (±SEM). (**B**) The two MenSCs populations demonstrate trilineage differentiation potential. Images

are representative of the differentiation potential of two donor samples. Scale bars are shown in white.

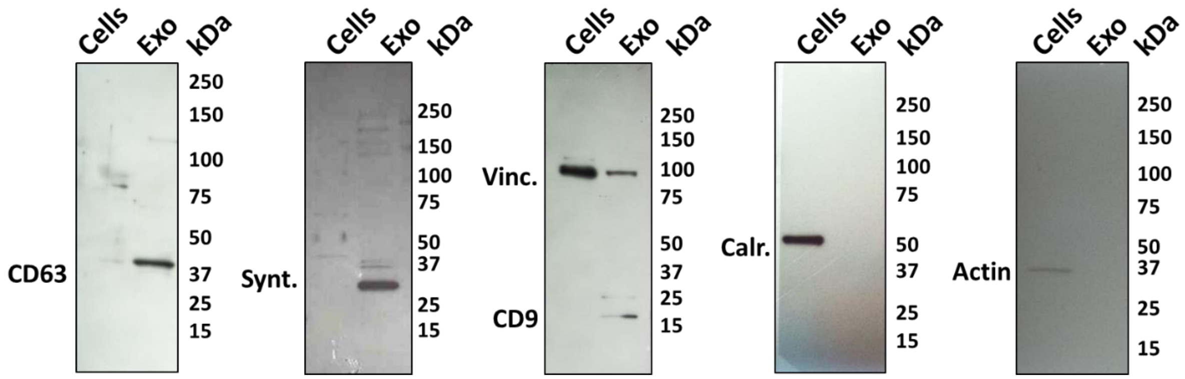

Figure S2: Full length western blots of figure 1. 15 µg of cell lysate (Cells) or exosomes (Exo) were loaded and positive markers CD63, Syntenin and CD9 as well as negative markers Vinculin (Vinc.), Calreticulin (Calr.) and β-actin (Actin) detected. The molecular weight marker in kilo daltons (kDa) is indicated for each blot.

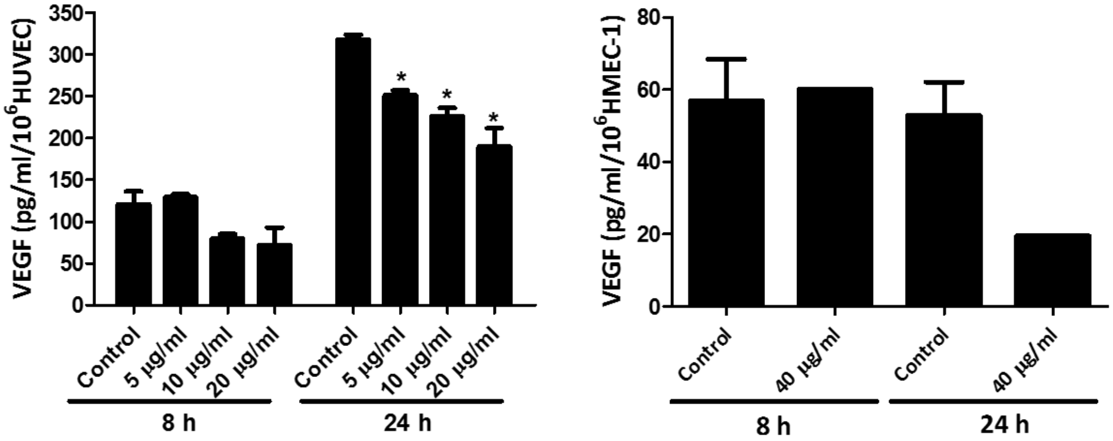

Figure S3: VEGF levels after 8h exosomes treatment analyzed using ELISA. Left panel: VEGF secretion by HUVEC after 8 and 24 h treatment with 5, 10 and 20 µg/ml MenSCs-exosomes quantified using ELISA. Right panel: VEGF secretion by HMEC-1 after 8 and 24 h treatment with 40 µg/ml MenSCs-exosomes.

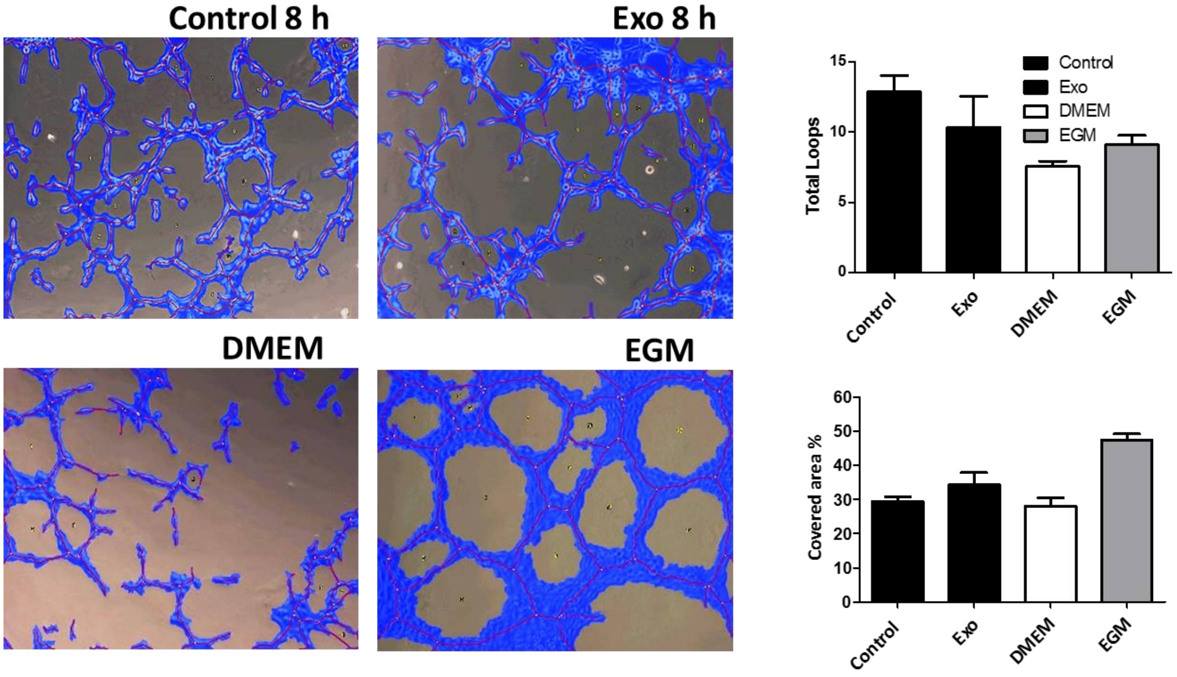

Figure S4: Eight hour exosome incubation is not sufficient to inhibit angiogenesis *in vitro*. Representative photographs of HUVEC tube formation on matrigel *in vitro*, using 8 h HUVEC supernatant without (control) and with 20 µg/ml exosomes, magnification 10x. The negative and positive technical controls were DMEM and EGM, respectively. Right panel shows total loops and percentage covered area quantified based on photographs using Wimasis.

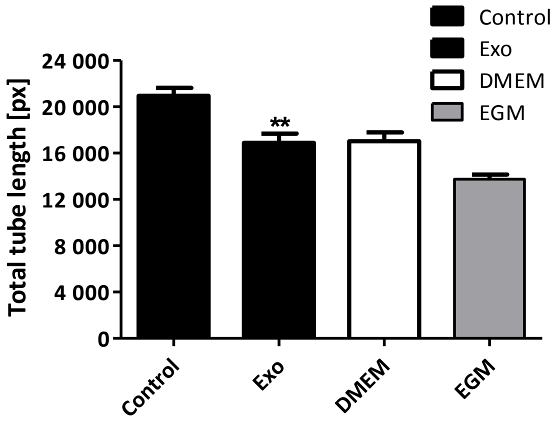

Figure S5: Total tube length of *in vitro* angiogenesis assay was quantified using Wimasis.

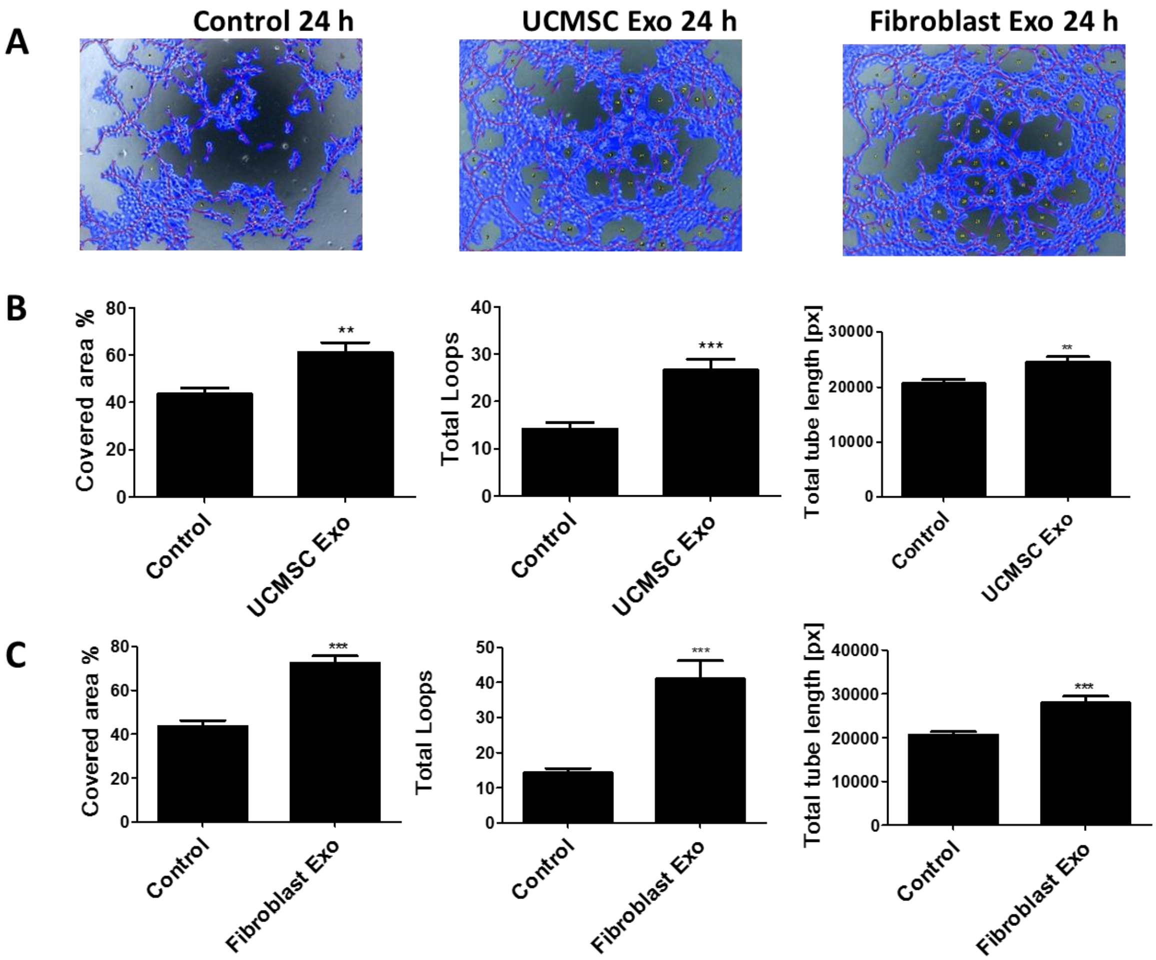

Figure S6: Tube formation using UCMSC and fibroblast exosomes. (A) Photographs of tube-formation using HUVEC conditioned medium of 24h control and with 20 µg/ml of UCMSCs- or fibroblast-exosomes, magnification 10x. (**B**) Quantification of percentage covered area, total loops and total tube length comparing control and UCMSCs-exosome-treated HUVEC. (**C**) Quantification of percentage covered area, total loops and total tube length comparing control and fibroblast-exosome-treated HUVEC.

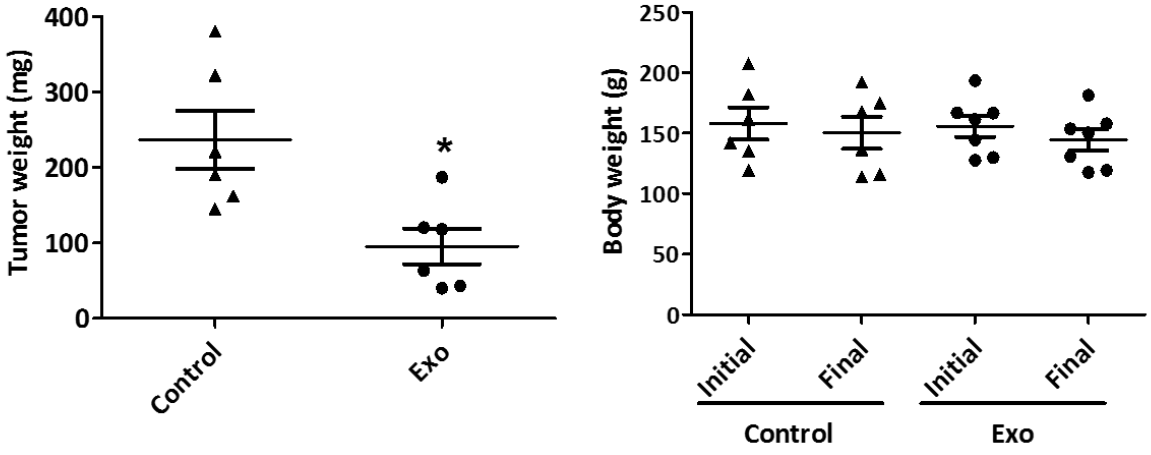

Figure S7: Exosome treatment significantly reduced tumor weight treatment without affecting body weight. Left panel: Average tumor weight of control and treated tumors at the experimental the end-point. Right panel: Body weight of treated and control hamsters at the beginning and end of the treatment.

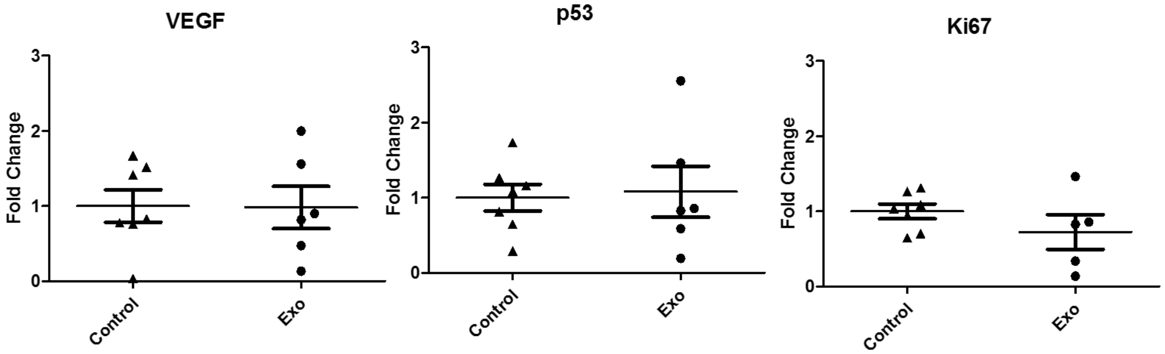

Figure S8: MRNA levels of VEGF, p53 and Ki67. QPCR realized for genes Ki67, p53 and VEGF from total RNA isolated from tumors at day 25 (experimental end-point).

Supplementary table S1: Primers used for qPCR

Gene	Primer	primer sequence	amplicon size	TM	GeneBank accession number	(ref)
**Ki67**	S	TGTATCCTTTGGTGGTCGTCTA	120 pb	77 °C	XM013111294	Ye et al. 2015[1]
	AS	GCTGGAGTGTGAGTGGTGAG				
**p53**	S	ATGCCGAATACCTGGATGAC	165 pb	82 °C	NM001281661	Ye et al. 2015[1]
	AS	GCGTGATGATGGTGAGGATA				

**VEGF**	S	CAGGAGTACCCCGATGAGATAGA	68 pb	77 °C	AF297627	Zivcec et al. 2011[2]
	AS	CCCCCACACCGCATCA				
**B2M**	S	GGCTCACAGGGAGTTTGTAC	77 pb	78 °C	X17002	Zivcec et al. 2011[2]
	AS	TGGGCTCCTTCAGAGTTATG				

Supplementary Methods

Tri-lineage differentiation

MenSCs cultured in 2D or with the Bionoc carriers (3D) were evaluated in their capacity to differentiate to mesodermal lineages by using the StemPro Differentiation Kits (Gibco, Grand Island, NY, USA) in accordance with manufacturer's instructions. Oil Red O, Alizarin red and Safranin O staining, respectively, determined adipocyte, osteocyte and chondrocyte differentiation, as previously described (Alcayaga-Miranda et al, 2012).

Immunophenotypic characterization of MenSCs

Immunophenotyping of MenSCs, cultured in 2D or with the Bionoc carriers (3D), were performed by flow cytometry after staining the cells with specific monoclonal antibodies CD73, CD90, CD105, HLA-DR, CD14, CD19, CD45 and CD34 (all from BD Biosciences, San Jose, CA, USA) using standard protocol. In addition, LIVE/DEAD®Fixable dead cell stain kit (Invitrogen, CA, USA) was used to determine the viability of cells according to the manufacturer's procedure. In all experiments, fluorescence-minus-one (FMO) controls were used to discriminate between the negative and positive population. The data were acquired using a FACSCanto II cytometer (BD Biosciences, San Jose, CA, United States) and analyzed with the FlowJo software V10 (Tree Star, Ashland, OR, United States).

Exosome Purification and Characterization

The exosomes were purified from MenSCs supernatant using sequential centrifugations as described previously[3–6]. The exosome pellet of the 100.000 G centrifugation was resuspended in PBS and stored at -80° Celsius (°C) until use. Exosome quantification was performed using bicinchoninic acid (BCA) protein assay kit (23225, Thermo Fisher) for protein content and nano particle tracking analysis (Nanosight NS300, Malvern Instruments, Malvern, UK) for particle concentration and size distribution. To visualize exosomes in an electron microscope, they were counter-stained with uranyl acetate for one minute and loaded on a formvar/carbon grid with copper mesh for electron microscopy (Ted Pella, No.01753-F). Image of exosomes were taken at 43,000 to 60,000 x magnification using the transmission electron microscope Philips Tecnai 12 Biotwin, at 80 kV. Exosomal markers were detected using western blot as described previously[4]. Briefly, 15 µg of exosomes or lysate were loaded on a 4-20% Polyacrylamide gradient gel and transferred to a polyvinylidenfluorid (PVDF) membrane (0.45 µm, RPN303F, GE Healthcare, Little Chalfont, UK). Antibodies against CD63 (1 µg/ml, sc-15363, Santa Cruz Biotechnology, Santa Cruz, USA), Vinculin

(1 µg/ml, ab129002, Abcam, Cambridge, UK), CD9 (1 µg/ml, ab65230, Abcam), Syntenin (1 µg/ml, ab19903, Abcam), Calreticulin (1 µg/ml, ab22683, Abcam), Actin (1 µg/ml, ab8227, Abcam) and HRP-coupled secondary antibodies goat anti rabbit and goat anti mouse (1:40,000, Biorad, Hercules, CA, USA). The bands were visualized using chemiluminescence reagent (34080 SuperSignal West Pico, Thermo Fisher, Waltham, Massachusetts, USA) and hyperfilm (28-9068-36, GE Healthcare, Little Chalfont, UK).

Cytotoxicity Assay

Cytotoxicity of exosomes on endothelial cells was quantified by lactate dehydrogenase (LDH) release into the supernatant using the CytoTox-ONE Homogeneous Membrane Integrity Assay (G7890, Promega, Madison, Wisconsin, USA) as recommended by the manufacturer.

ELISA

Secreted VEGF from endothelial cells was quantified using enzyme-linked immunosorbent assay (ELISA) (DY293B, R&D, MN, USA) as described by the manufacturer and VEGF contents were normalized according to the number of cells counted at the end of the experiment.

Tube formation Assay

Tube formation assays using HUVEC and HMEC-1 cells were performed as described previously[3]. In short, HUVEC ($4x10^4$) or HMEC-1 cells were resuspended in the respective conditioned media of exosome-treated and control cells and seeded on top of a matrigel layer (354230, Corning, New York, USA). After 4 h, the wells were photographed to quantify the tube-formation with Wimasis WimTube software (Wimasis, Onimagin Technologies, Cordoba, Spain). Please note that we used the same image for the EGM control in figure 4 and supplementary figure S4 since both time points were run in parallel using the same EGM control.

Gene expression analysis

Total RNA was isolated from buccal pouches using RNEasy Plus Mini Kit (Quiagen). Contaminating genomic DNA was degraded with DNAse RQ1 (Promega). One µg of RNA was reverse transcribed for 60 min at 42°C using 200 U M-MLV reverse transcriptase (Invitrogen) and 0.5 µM oligo-dT primers (Invitrogen). Real time PCR was performed in a final volume of 10 µL containing 50 ng of cDNA, PCR LigthtCycler-DNA Master SYBRGreen reaction mix (Roche), 3mM MgCl2, and 0.5 µM of each specific primer using a Light-Cycler thermocycler (Roche). To ensure that amplicons were from mRNA and do not from genomic DNA amplification, controls without reverse transcription were included. Amplicon validation was performed based on its size and melting point (supplementary table S3). To normalize data, β2 microglobulin was used as reference gene. The mRNA level of a target gene was calculated using the 2-ΔCt method and graphed as fold of change versus untreated group.

References

1. Ye, H., Yang, K., Tan, X. M., Fu, X. J. & Li, H. X. Daily rhythm variations of the clock gene

PER1 and cancer-related genes during various stages of carcinogenesis in a golden hamster model of buccal mucosa carcinoma. *Onco. Targets. Ther.* **8,** 1419–1426 (2015).

2. Zivcec, M., Safronetz, D., Haddock, E., Feldmann, H. & Ebihara, H. Validation of assays to monitor immune responses in the Syrian golden hamster (Mesocricetus auratus). *J. Immunol. Methods* **368,** 24–35 (2011).

3. Alcayaga-Miranda, F. *et al.* Prostate tumor-induced angiogenesis is blocked by exosomes derived from menstrual stem cells through the inhibition of reactive oxygen species. *Oncotarget* **7,** 44462–77 (2016).

4. Lopez-Verrilli, M. A. *et al.* Mesenchymal stem cell-derived exosomes from different sources selectively promote neuritic outgrowth. *Neuroscience* **320,** 129–139 (2016).

5. Thery, C., Amigorena, S., Raposo, G. & Clayton, A. Isolation and characterization of exosomes from cell culture supernatants and biological fluids **Chapter 3,** Unit 3.22 (2006).

6. Lopez-Verrilli, M. A., Picou, F. & Court, F. A. Schwann cell-derived exosomes enhance axonal regeneration in the peripheral nervous system. *Glia* **61,** 1795–1806 (2013).
